# Barriers to end-of-life care in an Oklahoma tribal community

**DOI:** 10.3389/fpubh.2026.1931261

**Published:** 2026-10-26

**Authors:** Lori L. Jervis, Meena Ramadugu, Bobby R. Saunkeah

**Affiliations:** 1 Department of Anthropology and Center for Applied Social Research, University of Oklahoma, Norman, OK, United States; 2 UT Southwestern Medical Center, Dallas, TX, United States; 3 Chickasaw Nation Department of Health, Ada, OK, United States

**Keywords:** American Indian, barriers, end of life, hospice, palliative care, tribal health system

## Abstract

American Indians/Alaska Natives (AIANs) utilize end-of-life (EoL) services at lower rates than other U.S. populations, despite evidence that hospice and palliative care improve quality of life and reduce suffering. This study explores barriers to EoL care within the Chickasaw Nation through semi-structured interviews and focus groups with 27 participants, including health care providers, tribal leaders, and family members of dying persons. Findings reveal pervasive lack of knowledge and misconceptions about hospice, the association of hospice with imminent death, and psychological barriers such as death anxiety and denial. Cultural and generational imperatives that family should provide care and reluctance to allow outsiders—especially non-tribal citizens—into the home further impede service uptake. Systemic gaps, including the absence of tribally operated hospice programs, compound these challenges. Bureaucratic insistence on a 6-month timeline to death, when juxtaposed against the desire for a cure, poses another seemingly intractable barrier. While local non-tribal hospice agencies were generally viewed as high quality and culturally accommodating, participants nonetheless expressed a strong preference for hospice services integrated within the tribal health system and responsive to local culture. These findings underscore the need for culturally responsive education and tribal involvement in EoL care delivery.

## 1 Introduction

American Indians/Alaska Natives (AIANs) utilize end-of-life (EoL) services at lower rates than other US populations (1–4). These services (e.g., hospice and palliative care) have been associated with improved quality of life and greater life expectancy (5–7), as well as better pain relief, superior symptom control, reduced patient anxiety, and fewer hospital admissions (8)—particularly when EoL services include educational interventions (9). Caregivers also benefit from these services through strengthened psychosocial support, improved communication, and reduced emotional distress (10). That EoL services are not reaching much of the AIAN population increases the likelihood of unnecessary suffering in the final days of the terminally ill and their families (11).

Barriers to accessing EoL care are many, spanning systemic, financial, psychosocial, and cultural factors. In the US, barriers to hospice include misconceptions about financial and eligibility requirements, challenges navigating highly bureaucratized healthcare systems, beliefs that hospice requires relinquishment of all care, underutilization due to minority status, and difficulty discussing and accepting mortality (12–16). Reimbursement concerns among providers may mean some patients are not offered hospice in the first place. An online randomized survey of 495 U.S. hospice clinicians and administrators involved in admission decisions found that hospice clinicians were significantly less likely to admit patients requiring complex high-cost treatments (17). Emotional and psychological factors, such as grief, anxiety and the stigma of “giving up” on life can also prevent patients and families from engaging in EoL care (18, 19).

For AIANs, multiple systemic and cultural barriers to EoL exist (20, 21), including fragmented hospice delivery systems (Soltoff et al., 2022), a scarcity of tribally operated and Indian Health Services hospice and palliative care programs (22), lack of access to health insurance (23), and difficulty accessing care due to geographic remoteness (23, 24). AIAN health care delivery systems frequently lack the clinical workforce and funding necessary to deliver hospice services that meet Medicare and Medicaid requirements, forcing many AIAN patients to rely on external programs that might not align with cultural values or expectations around death and dying (25). Misunderstandings of hospice (26) and low levels of cultural awareness or sensitivity among care providers (23, 26) create additional barriers.

Rural barriers to hospice care compound the already significant challenges faced by AIAN populations. Numerous barriers to EoL care exist in rural areas, including fragmented medical systems, provider shortages, financial/structural barriers, lack of EoL knowledge and awareness among both service users and providers, and patient concerns/misinformation (27, 28). Patients in rural areas experience additional logistical barriers, including long travel distances and transportation challenges (29). These issues are exacerbated by a national shortage of palliative care clinicians, particularly in rural areas, and by limited knowledge of hospice eligibility and benefits among both providers and patients (30).

This article focuses on barriers to EoL care within an Oklahoma tribe, drawing on semi-structured interviews and focus groups with 27 healthcare providers, community leaders, and family members. At its heart is understanding how EoL care is understood locally, barriers to its utilization, and what might facilitate access to care.

## 2 Methods

This study built upon a 2019 pilot study on EoL service availability and access in tribes across the US, funded by the University of Oklahoma's Stephenson Cancer Center. A telephone survey of 52 service providers/administrators of tribal elder programs, supplemented by three focus groups with hospice care providers from two nonprofit, non-tribally owned hospices in Oklahoma that served American Indian clients, pointed to numerous economic and infrastructural barriers as well as a basic lack of awareness and knowledge about EoL services. The vast majority of tribes provided no EoL services at all, with family members and non-tribal hospice services providing the bulk of care for terminally ill people (31). Reluctance to utilize hospice was widespread, and admission was often delayed until patients were actively dying, too late to benefit from all that this service offers. Based on these broad findings, we wanted to better understand how lack of tribal hospice care and attitudes toward EoL services manifested at the level of a local tribal community.

Prior to data collection, the project (funded by the University of Oklahoma Stephenson Cancer Center via a National Cancer Institute Cancer Center Support Grant P30CA225520) underwent an extensive approval process by both the participating tribe's Institutional Review Board and the University of Oklahoma Institutional Review Board. Before commencing data collection, the team met with members of the Chickasaw Nation Tribal Health system to ensure that project questions and methods were responsive to the local community. Data collection occurred between 8-2019 and 5-2020. In spring 2020, the Covid-19 pandemic disrupted data collection, making in-person interviews impossible. The final two interviews were conducted via telephone to minimize risk of disease spread. Data collection ended prematurely due to the crisis the pandemic posed to the Chickasaw Nation Health System, as to health care systems nationally and globally, as attention turned to the emergency conditions wrought by the pandemic.

A total of 27 individuals participated in this study, the majority health care providers employed by the tribal health care system (*n* = 22, 81%). Seventeen of these participants were participants in two focus groups with Community Health Representatives (CHRs). These focus groups were held in conjunction with CHR team meetings in order to minimize burden related to travel across the large rural area in which CHRs work. Ten participants, recommended by community partners for their knowledge of key issues related to EoL care in the Chickasaw Nation, participated in individual semi-structured interviews. Of the 10 interview participants, five identified as health care providers (e.g., doctor, nurse, social worker, and chaplain), three tribal leaders, and two family members of persons who had died. Many participants had experienced hospice in multiple roles (e.g., in both their professional capacities and personal lives). Due to their overall greater familiarity with hospice, participants generally believed themselves to have considerably more knowledge of EoL care than the larger tribal community. Most participants identified as female (*n* = 22, 81%) and American Indian (*n* = 20, 74%). All non-Native participants were CHRs, six of whom identified as white and one as Latina. All participants are referred to by pseudonyms.

Interviews were conducted by three researchers, all of whom had extensive experience in tribal contexts, and one of whom is a citizen of another Oklahoma tribe. In both focus groups and interviews, participants were asked about the community's familiarity with EoL services, experiences with and beliefs about EoL and hospice care among tribal citizens, barriers to obtaining EoL care, what EoL needs existed in the community, how things could be improved for dying people and their families, and what they would most like to see in a tribal EoL program. Focus groups and interviews were audio recorded and transcribed verbatim. Interviews were analyzed using inductive thematic analysis with the assistance of NVivo 15 (32, 33). The interview team initially reviewed data for emerging themes. A codebook was developed that included themes found in the data, such as perceptions of EoL care, caregiver experiences, and death denial. These themes were analyzed in more detail in a second round of focused coding. One researcher was primarily responsible for coding the data (MR), while a second researcher (LJ) reviewed the coding to identify and resolve potential issues or discrepancies. Discussions with community partners throughout the project provided additional validation of findings.

## 3 Setting

As the former “Indian Territory” to which American Indians were forcibly removed in the 1830s (34), the southcentral state of Oklahoma is today home to 38 federally recognized tribes. The Chickasaw Nation, the tribal nation which is the site for this study, is large, its treaty territory encompassing 7,648 square miles. The tribe includes nearly 83,000 citizens, most of whom live in Oklahoma. The tribe is economically successful and operates its own award-winning tribal health care system (2024 Baldridge Award, 2024 CMS 5-star ranking, one of America's Best in State Hospitals 2024), rather than receiving direct health services from the Indian Health Service. While the tribal health system is highly reputed, the state of Oklahoma overall is characterized by poor health status, severe health disparities, and a large number of Health Professional Shortage Areas (35–37).

While offering comprehensive health services, the tribal health care system does not include hospice or EoL palliative care. As common among many tribes (31), EoL care is largely outsourced as is common to local nontribal home health care agencies. Informally, some EoL-adjacent services are provided by the tribe's Community Health Representative (CHR) program, paraprofessional community health workers (38, 39). Within the Chickasaw Nation, CHRs are expected to provide very little direct care and to focus mostly on patient transportation, patient advocacy, liaison for tribal/community resources, patient education and health promotion/disease prevention. This work, which often includes lengthy trips to health care providers, puts CHRs in close proximity to patients where they are able to gain insights into their perspectives. CHRs made it clear that because many of their clients are older and have no family to help them, they often end up providing “a lot more than transportation,” including companionship and help with household tasks.

## 4 Findings

Interviews with participants pointed to several factors that decreased community members' likelihood of accessing EoL services, including lack of knowledge, misinformation, and the intense desire for curative care. They also identified strengths within the current health care service economy that could be harnessed to increase utilization, namely confidence in the tribal health care system and the availability of high-quality local hospice services.

### 4.1 Knowledge of hospice and death anxiety

Perhaps the greatest barrier to receiving EoL care, according to participants, is the community's lack of knowledge about hospice and palliative care. This lack of knowledge was believed to be ubiquitous in the tribal community and was considered so extreme as to be effectively non-existent. Participants viewed this as especially problematic since prior experience with EoL care was believed to increase the likelihood of service uptake. In this respect, participants differed greatly from the broader community, as many had experience with hospice and viewed it positively. In contrast, the larger tribal community, despite knowing next to nothing about hospice, was thought to view it negatively, with one doctor referring to it as a “stigma.” This negativity derived from the perception that hospice is “a death sentence.” Community members were said to believe that accepting hospice would actually expedite death.

Given their own experiences with hospice, participants saw these community notions as misconceptions. Jane, a nurse who frequently worked with patients with cancer, tried “to explain to them that that is not what this is, that we have had patients on hospice for months.” Some participants, such as Kristy, a tribal citizen who had lost several family members to cancer, had come to believe that surviving hospice was definitely possible: “I used to think when I heard the word “hospice” that it was terminal, that the person was close to end of life at any time. But in past dealings I have come to learn that you can have hospice in, and when they get better, then hospice goes away.” This perspective, however, had not permeated the wider community which continued to associate hospice with imminent death.

But despite the fact that hospice utilization does not in fact always equate to rapid death, admission to hospice requires at least some acceptance that death is probable. At minimum, it requires the reality of death to be moved into conscious awareness of patient and family. This kind of frank conversation may be at odds with the usual ways that death is handled. Bonnie, a health care administrator who had worked as a hospice social worker in the past, remembered that in her family no one talked about dying: “A lot of times it really wasn't discussed. It was something that just happened….We all knew grandma was sick so we all just took care of her and that was it.” Speaking out loud what normally goes unspoken arguably makes death denial more difficult to sustain. A CHR named John recalled how a client's husband refused hospice because he didn't want to believe that his wife had only a short time to live. In the end, the wife died quickly anyway. “He was just like blown away cause they thought it would come out to six months. And he just couldn't believe she was gone in less than a week.” Accepting death as inevitable and impending was a truly tough pill to swallow, and constituted a major barrier to accepting hospice services.

### 4.2 Giving up and the quest for a cure

To qualify for Hospice under Medicare, which pays for the majority of hospice care in the US (40), a patient must be certified as terminally ill by a doctor (i.e., having a life expectancy of 6 months or less), and must agree to a palliative rather than curative care approach (41). This too is a tough pill to swallow. Jean, a doctor whose own uncle benefited from hospice care, observed that patients “want to explore all options and keep going until they really see that this is a terminal diagnosis.” Jean noted that patients “are not ready to accept the fact that this is just not going to get better” and “want to think that uncurable things can be cured if they keep going in the direction of aggressive medicine….Some of it is just clinging to an idea that this is going to be cured, that this going to get better, whatever the diagnosis is.” This tendency is exacerbated by the culture of medicine itself, which trains doctors to “charge into battle…and treat, treat, treat,” in the words of a doctor named Courtney. The medical imperative to cure, coupled with patients and families' death denial, is a potent brew that kept the dying on the path of futile and often painful curative care.

Against the backdrop of intense longing for a cure, participants declared that hospice was seen as “giving up” within the community. One CHR, Mandy, contrasted the “comfort care” of hospice with “*care,”* meaning that “If it's palliative, I'm going to make you comfortable, but I'm not going to give you the care to try to make you better.” This statement suggests that hospice may be viewed by some as partial vs. full care. Both patients and families found this possibility very difficult to accept. “It's like it's the end and they don't want to give up,” a CHR named L'Jonna explained, “So they don't want hospice in.” Jean, the doctor, went further, noting that people see hospice as not pursuing *any* additional treatment. This means not only relinquishing the search for a cure, associated with loss of hope and even despair (42), but signals a more complete abandonment of care.

Darren, a Christian minister, illustrated how sometimes faith itself could complicate acceptance of the inevitability of death. As a close relative lay dying, Darren found himself insisting “that God can still heal….My faith was still saying, ‘Maybe, just maybe, this will turn around.' ” Reflecting on that experience, Darren observed that hospice personnel “work with this all the time,” and while they may think, “This isn't turning around,” they allow patients and families to work through the process in their own way rather than chastising them. He remembered hospice staff calmly telling him, ‘Okay, but here is what we are going to do,' and [sticking] to their plan.”

Eventually some patients, especially those who had suffered through a long illness, were ready for death. Family members, on the other hand, were believed to be slower to come around to this acceptance. Jean the doctor recalled that:

“We've had many occasions where the patient knows what they want. They know they are close to the end of their days and they are ready for it, but the family is not ready for it and so the idea [of hospice] just kind of limps along. They come back in [for more aggressive care] to try and appease their family when what they would really prefer is hospice, and it just kind of becomes continual conversations until everyone is ready to accept what it is the patient wants.”

The deep desire to be cured and to forestall death delays hospice admission, resulting in short hospice stays which prevent patients and family from fully benefitting from the program (43, 44). Short hospice stays, in turn, no doubt merely reinforce the idea within the community that hospice brings imminent death, a self-reinforcing cycle.

### 4.3 End-of-life care preferences and tribal identity

The overwhelming preference among community members is to die at home, and for most this was said to be possible. Dying at home is seen as preferable due to “the surrounding of people that would be with them. And it's just the feeling of security being at home,” noted Kristy, who had lost several family members to cancer. It is also “easier on their families,” observed Jane the nurse. “My patients always seem to worry about what is easiest for their families.” Those who died at home were generally cared for by family members with the assistance of local home health agencies. The requirement that patients have a family caregiver is thought to constitute yet another barrier to hospice admission, as some families are logistically or financially unable to fulfill that role. Indeed, one of “the biggest misconceptions that [patients and family] may have is that once they are in hospice they are going to have somebody [from the agency] there, but they are not there all the time,” observed Kim, a social worker who had a positive experience with hospice when her grandmother was dying. She continued: not everyone has “family members who could be at the patient's beck and call.” Those without family support were generally cared for in local nursing homes or the tribal hospital, the latter said to be far from ideal due to the financial cost it imposes on the tribal health care system.

While not everyone can die at home, the belief that care *should* be provided by the family is near universal in the community. Jean the doctor described this as “a big generational and cultural thing, but especially in the older tribal members.” The prevailing reasoning is that “Family is supposed to take care of you. You are not supposed to have an outside person come in and take care of you. So my family can take care of me, I don't need [hospice].” Jean noted that “a lot of our older patients don't want anyone [who is not family] in their home.” This aversion sometimes extended to not wanting outsiders of *any kind* there, especially non-Natives. Missy, a CHR, proclaimed that “Down in my area, you‘ve got the certainty that if they don't think you're Native, they don't want you in their house to begin with,” especially “when it comes to the time that they have to have hospice.” Sam, also a CHR, explained that “A lot of them that are passing now, they want Indians coming in.” One older client was described as not wanting non-Native people in her home that she didn't know: “Different people coming in and out of your house….She just didn't want it. The family just went ahead and stayed and took care of her needs.”

While the dying are strongly believed to not want outsiders in their homes, the root of the problem is fuzzy, with participants sometimes pointing to non-family members as the primary concern, sometimes to non-Natives, and at other times simply to strangers of any kind. Some participants highlighted modesty and the desire for privacy among clients and family members, while others were said to fear that outsiders (and sometimes specifically “white people”) would judge them for “how the family lives” or the condition of their home. As Darren the minister put it, “They are somewhat embarrassed of that, [thinking] these people are going to come into my home and [now] I need to go home and clean this room out….Some people are like ‘These people are coming in and are they going to be judgmental about how I live?”

Despite these concerns, EoL care is in fact outsourced to non-tribal home health and hospice agencies run largely by non-tribal citizens. Based on their experiences, participants overwhelmingly saw these agencies as both readily available and quite good. Kristy reflected on family members who had died with the assistance of local hospice care: “They‘re very helpful, very compassionate, when they're in your home and taking care of your loved one. I don't know what we did before we had hospice. They were lots of help to us.” These agencies were also said to accommodate Indigenous cultural and spiritual practices. Darren, a minister whose immediate family member had been a hospice client, stated that these providers “Don't push. They come in and they kind of lay it out step by step and try to stick to that plan and….if there are boundaries, let's find out what they are, and come in with a sense that I am here to help you through this.” While the majority of hospice providers were white, participants felt they were nonetheless generally able to overcome barriers of trust among patients and families.

The fact that hospice typically does not maintain much of a presence in the home until the patient is relatively close to death complicates the establishment of this trusting relationship, said to make it more difficult to “make a bond” with a tribal elder. Sometimes a CHR was called in to introduce a non-Native provider to patients and families. A CHR named Lily explained that “you‘ve got to be that bridge between the patient and the [hospice] company that's there, because a lot of times they just look at the company like they're just here for the money,” rather than being primarily concerned with the patient. As Darren the minister explained, “[Hospice] is a business, and nobody wants you to come in and take of their loved one in a business-minded way.” CHRs understood their role as trying to make the patients and families comfortable with outside providers: “So basically, we start out, ‘Hey, you can trust them. They‘re OK.' And then after a while, they eventually get used to them. They're comfortable, they trust them.”

Despite generally positive assessments of local hospice services, a few participants saw these agencies as lacking in cultural sensitivity. The most common complaint was that hospice providers do not always understand that they need to involve the full extended family. Bonnie, a health care administrator who had worked as a hospice social worker in the past, recounted how, when her sister was dying, she felt disrespected because hospice honored the preferences of her nieces and nephews over her own. “With Indian families you are talking about family. Not just the ones that live in that household, but you are talking about aunts and uncles, whoever else wants to be there,” she explained, “a room full of people.” Yet other participants did not construe the tribal culture as a barrier to hospice utilization, with Sam the CHR explaining that “in Oklahoma we do not have as much cultural immersion as we see in like New Mexico and Arizona.”

One area in which the topic of culture was explicitly evoked is in the context of religion. Christianity is the predominant religious orientation of the community, and some participants want Christian preachers to “come in and say a prayer for them,” as Mel the CHR noted. Some dying individuals want what Sally the CHR described as “natural traditional healing” in addition to conventional medical care, but this is in short supply in the tribe. Darren the minister lamented that there are very few traditional practitioners, such as medicine men, left in the tribe. “I knew one in high school and I think he passed away probably about 15 years ago and so we don't really have those resources no more. We still have some people who pray with the sage and the sweetgrass, different things, and their prayers are the traditional prayers that have been handed down, but outside of that the spiritual part of it is not there anymore.” Indigenous practices are limited to the “use [of] sage to kind of cleanse and to kind of send prayers up,” stated Patrick, a tribal leader.

Despite and perhaps because of the loss of some traditional practices, formal tribal services may take on special importance. To this end, participants wished for a tribally operated hospice program. This was not based on dissatisfaction with the local hospice services, but with a general sense that like other types of health care, this care too should be delivered by the tribe. While a few health care providers believed that providing EoL services within the tribe might reduce bureaucratic barriers and increase cultural sensitivity, this was not the driving factor for most participants. Simply put, the tribal health care system is what community members know and are comfortable with, and there is a general expectation that all care be provided there. Patrick, a tribal leader, put it this way: “[It's] the community telling us “We would rather have the tribe do this for ourselves, have Chickasaws taking care of Chickasaws. Our health system taking care of this community.” Moreover, Patrick noted that there is a general expectation in the tribe that “You want to complete the circle….[have] a system that takes care of you from birth to death.”

## 5 Discussion

This research examined perceptions of and use of EoL care in an Oklahoma tribal community. The finding that knowledge of hospice and palliative care is minimal and plagued by misconceptions is shared by many US demographic groups, both tribal (45–47) and nontribal (13, 19, 29, 48). Death anxiety and denial too, are hardly unique to this tribe, posited to be an inherent feature of being human (49). Likewise, the expectation that family should provide care is commonplace if not always perfectly fulfilled (50). Thus, citizens of this tribe share many common concerns related to death and dying with those of other communities. The tendency to divide others into insiders and outsiders is likewise prominently featured across human communities, and has been found in situations that involve careworkers from other cultures providing care within the home (51). The aversion to those deemed as outsiders coming into the home, combined with lack of knowledge of hospice and its stigma, as well as the tendency of hospice providers to offload care to family members until death is imminent, meant that family did in fact provide the bulk of EoL care. The formal EoL services tribal citizens received were provided by outside agencies staffed by the very outsiders that patients and families were believed to not want in their homes.

Despite these challenges, non-tribal hospice agencies were believed to be able to overcome many of these barriers and provide high quality and mostly culturally congruent care. Even so, participants wanted EoL care to be housed in the tribal health care system. Rather than being driven by a serious problem in the extant system, this could be viewed as wanting to make something good even better. Hospice care, believed by participants to be an overall positive, might be improved if it were part of the tribal health system community members know and trust.

There are 574 federally recognized entities across the United States (52), and the Chickasaw Nation is only one among them, meaning these findings cannot necessarily be generalized to any other tribe. Still, our findings do echo those of other rural American Indians (15, 30, 53). Previous research documents mistrust of health systems among American Indians in general (54–56), along with perceptions of poor care (57). The observation that patients and families did not want “outsiders” in their home, coupled the need for CHRs to foster trust in extant private hospice services, bolsters the justification for embedding hospice within the generally trusted tribal health system. Putting hospice within a nonprofit system would also counteract concerns that the profit motive of hospice might overrule its ability to provide care.

But moving hospice into the tribal health system does not resolve one of the greatest barriers to access, the problematic relationship between official requirements for hospice admission and the universality of death anxiety and death denial (49), testified to by the vast literature on mortality salience and terror management theory (58–61). Admission to hospice requires accepting the unfathomable and intolerable fact that one is dying and abandoning the quest for a cure, equated with hope. That is, accepting hospice means giving up hope as conventionally construed, suggesting the need for a more expansive conceptualization of hope. It is likely that providers and the Medicare program itself severely underestimate the psychological challenge imposed by the requirement to give up curative care. The aversion to death and desire to extend one's lifespan is incompatible with hospices' 6 months-to-live timeline and the imperative to give up on a cure (62). The inability to reconcile these contradictory requirements and impulses, compounded with mistrust and misinformation, too often leads to ineffective and overly aggressive care, as well as a shortened course of hospice which reinforces the equation of hospice with imminent death. It is a missed opportunity to alleviate unnecessary suffering. More flexibility in Medicare's guidelines would be ideal, but in the meantime practitioners may consider framing the 6 month timeline as a bureaucratic requirement that allows one to obtain important services rather than prognostication of a client's actual death date.

Improving EoL care in tribal communities requires strategies that are both culturally responsive and attentive to psychosocial and systemic barriers. Educational initiatives must address community misconceptions about hospice, emphasizing its benefits while acknowledging the very real barrier posed by death anxiety and denial. Capitalizing on the role of CHRs as cultural intermediaries to outside hospice agencies, while integrating a suite of desired EoL services within the tribal health system, would expand options for tribal citizens, especially those who strongly desire to be cared for by Native caregivers. Tailoring EoL services to the local community, rather than relying upon a generic Native American cultural adaptation, would further increase its beneficial potential.

## Data Availability

The datasets presented in this article are not readily available because they contain identifiable information. Requests to access the datasets should be directed to lori.jervis@ou.edu.
